# Mental Health and Psychological Wellbeing during the COVID-19 Lockdown: A Longitudinal Study in the Balearic Islands (Spain)

**DOI:** 10.3390/jcm10143191

**Published:** 2021-07-20

**Authors:** Joana Ripoll, Sara Contreras-Martos, Magdalena Esteva, Aina Soler, Maria Jesús Serrano-Ripoll

**Affiliations:** 1 Primary Care Research Unit of Mallorca, Balearic Health Service, 07002 Palma, Balearic Islands, Spain; sara.contreras.martos@gmail.com (S.C.-M.); magdalena.estevacanto@ibsalut.es (M.E.); aina.soler@ibsalut.es (A.S.); mariajesus.serranoripoll@ibsalut.es (M.J.S.-R.); 2 Balearic Islands Health Research Institute (IdISBa), 07120 Palma, Balearic Islands, Spain; 3 Primary Care Research Support Unit Costa Ponent, University Institute for Primary Health Care Research Jordi Gol i Gurina (IDIAPJGol), 08007 Barcelona, Spain; 4 Department of Medicine, University of the Balearic Islands (UIB), 07122 Palma, Balearic Islands, Spain; 5 Department of Psychology, University of the Balearic Islands (UIB), 07122 Palma, Balearic Islands, Spain

**Keywords:** prospective, lockdown, COVID-19, mental health, anxiety, depression, psychotropic drugs, psychological wellbeing, fear, life satisfaction, self-perceived health

## Abstract

Confining the entire population to a lockdown after the outbreak of SARS-CoV-2 was an unprecedented measure designed to protect the health of those living in Spain. The objective of the present study is to assess the evolution of mental health and psychological wellbeing during lockdown. To do this, we carried out a longitudinal study, via an online survey over the eight weeks of lockdown (weekly assessments). Sociodemographic variables were recorded, along with data related to COVID-19, psychological wellbeing (anxiety, depression, psychotropic drugs, consultations made to improve mood or anxiety), life satisfaction, and self-perceived health. A total of 681 individuals participated in the study, 76.8% were women; the mean age was 43 years old (SD = 12.7). Initially, high scores were reported for anxiety, depression, and the number of consultations to improve mood, but these decreased significantly over the study period. The reverse seems to be true for life satisfaction, perceived good health, and intake of psychotropic drugs. We also identified groups whose psychological wellbeing was more susceptible to the effects of lockdown. Women, those worried about their jobs after the pandemic, and those afraid of being infected were the most affected individuals. More generally, after the initial negative effect on psychological wellbeing, various indicators improved over the lockdown period.

## 1. Introduction

The COVID-19 disease, caused by the SARS-CoV-2-type coronavirus, surfaced in December 2019, in China. By March 2020, it was declared a pandemic by the World Health Organization (WHO) [1]. This is a highly contagious disease and has potentially serious symptoms, leading to large numbers of infected individuals and deaths [2]. Thus, various governments, under guidance from leading epidemiologists, took extraordinary measures to protect the health of their populations, reduce the spread of the virus, and avoid overwhelming healthcare systems. In Spain, the government declared a state of emergency on 14 March 2020 [3], implementing containment measures such as a lockdown, curtailing the free movement of individuals, implementing social distancing, closing essential services including schools and universities, and shutting down most other economic activities, such as restaurants, gyms, and shops, among others. This unprecedented situation, in which nearly three billion people around the world were subjected to some type of lockdown [4], led to worry about the potential consequences that this might have on people’s mental health [5]. Lockdown in Spain forced numerous people to remain in their homes, deprived of their freedom; it represents a situation that had never before been experienced, and we still do not know its real short- and medium-term psychological effects. Lockdowns might have an impact on emotional functioning and could lead to stress and symptoms associated with anxiety and depression [6,7]. Along this line, a study of such effects during a lockdown was carried out with 52,730 individuals in China [6], and it found that nearly 35% of those surveyed experienced anxiety. Another study of 3672 Italians found a prevalence rate of depressive symptoms of 28% [8]. The growing threat of the epidemic created a worldwide atmosphere of anxiety and depression due to social isolation, the disruption of travel plans, an overburdening of information by the media, and the hasty purchasing of basic necessities provoked by the panic [9]. Factors such as fear and worry may have had psychological implications and repercussions [10]. Additionally, being isolated disrupts social relationships, an important source of support for many people [11,12]. To all of this we must add the unease that comes with uncertainty regarding the future, one in which an unprecedented socioeconomic crisis seems to be looming [13].

The unique nature of the current situation makes it absolutely necessary to gather useful information for evaluating the impact of these measures and prevent the undesirable effects of an extensive lockdown of the population. The present study considers the evolution of the psychological wellbeing of the general population, as well as factors that contributed to its evolution, over lockdown.

## 2. Materials and Methods

### 2.1. Design and Participants

We carried out a prospective study with weekly follow-up via an online survey that was given out from the first week of lockdown to the last. Participants were adults (over 18 years old), and residents of the Balearic Islands (Spain). They were recruited using the snowball technique with the telephone contacts of the research team involved in the present project. Specifically, the study ran from 15 March (when a state of emergency was declared) to 10 May (when phase 0 of the de-escalation came to an end in the Balearic Islands).

A questionnaire was created using Google Forms, an online tool for preparing forms. The survey was sent from an e-mail account created ad-hoc to store the telephone numbers of the participants with restricted access to the principal investigator. This facilitated the management of the contacts and their subsequent deletion without leaving a trace on the device. Each week, once the responses had been downloaded from a spreadsheet, the online version of the file was deleted. Likewise, to guarantee the pseudo-anonymization of the surveys, the cell phone data were replaced by a code associated with each phone number. The questionnaire was sent out weekly over the course of eight weeks via the WhatsApp messaging app, and participants had a period of three days to respond. The first assessment (first survey) included an explanation of the content and objectives of the study, as well as information regarding data protection and the identification of who was to be in charge of the data that would be stored by the Servei de Salut de les Illes Balears (Ibsalut; the public healthcare service of the Balearic Islands). This assessment also highlighted that by answering the questionnaire, respondents were providing informed consent to participate in the study.

### 2.2. Outcome Measures

Sociodemographic variables: sex, age, level of education (grouped as university studies or no university studies), employment status (working, unemployed, or retired), average monthly income on a five-point Likert scale (1 = with difficulties to reach the month end, 5 = not any difficulties to reach the month’s end; grouped as: 1–3 = difficult to reach, 4–5 = easy to reach), household situation during lockdown (number and people living with relationship), as well as household characteristics (outdoors space and size).

Situational variables were defined at the base time and their evolution was monitored over the eight-week lockdown: job conditions (working on-site, from home, or mixed; those who worked from home for at least 75% of the period analyzed were considered to work from home); concern over employment status after lockdown, fear of COVID-19 infection, and interest in pandemic evolution were all measured on a five-point Likert scale (1 = little interest/worried, 5 = very interested/worried; those who answered 4–5 at least 75% of the time over the study period were considered to have high levels of interest or worry); and the number of times out of home during the week (none, 1–3 times, 4–5 times, or daily; those who went out of their homes three times or less during the week, over at least 75% of the period analyzed, were considered to have rarely gone out) grouped as rarely out and frequently out.

Dependent variables: indicators of psychological wellbeing: scales for symptoms of anxiety and depression, consumption of psychotropic drugs, and consultations to healthcare professionals or websites to improve one’s mood or reduce anxiety. Measurements were taken during the eight weeks of lockdown. Generalized anxiety was assessed using the generalized anxiety disorder (GAD-7) scale, which includes seven Likert items with responses between 0 and 3. The scale has high levels of validity and reliability [14], was adapted to Spanish, and approved for use in Spain [15]. Depressive symptoms were measured using a version of the patient health questionnaire (PHQ-9) [16] that has been approved for use in Spain [17] and which is made up of another nine Likert items with responses between 0 and 3. The consumption of psychotropic drugs to reduce anxiety or insomnia during lockdown was considered with a direct “yes or no” question. To assess consultations carried out with health care professionals or websites to improve mood or lessen anxiety, participants were asked to choose between three options: whether they had consulted a professional, whether they had visited a website, or whether they required neither.

Other variables: both life satisfaction, measured on a 0–10 Likert scale (0 completely unsatisfied and 10 completely satisfied), and self-perceived health (excellent, very good, good, regular, or bad) were measured over the eight weeks of lockdown.

Optimism regarding one’s personal future and optimism regarding the future of society after the pandemic was only collected on the final questionnaire (week 8). It was measured on a five-point Likert scale (1 = not optimistic at all, 5 = very optimistic).

### 2.3. Statistical Analysis

We carried out a descriptive analysis of the observed sociodemographic variables to identify the characteristics of our sample and to assess the overall evolution of each of the dependent variables each week during the study period (absolute frequencies and percentages). To analyze the differences in the indicators of psychological wellbeing from the first week to the fourth week, and from the beginning to the end of lockdown (first and eighth weeks), we carried out a paired analysis, using McNemar’s test. We used generalized estimating equation (GEE) analysis to examine weekly changes in psychological wellbeing indicator scores. The relationship of the sociodemographic and situational variables with the evolution of the indicators of psychological wellbeing halfway through and at the end of lockdown was assessed using the chi-squared test. For the analysis, we transformed some dependent variables. Variables referring to anxiety, depression, and the consumption of psychotropic drugs were recoded into three categories: unchanged, increase in symptoms or consumption, or decrease in symptoms or consumption, all between weeks 1–4 and weeks 1–8. The variable regarding consultations with a professional or a website to improve one’s mood or reduce anxiety considers whether a visit was made at least once between weeks 1 and 4 or whether a visit was made at least once between weeks 1 and 8.

## 3. Results

A total of 681 people responded to the first questionnaire. They were recorded anonymously as participants in the study and received weekly follow-up questionnaires over the eight weeks of the study. The average response rate during the first four weeks was 72% and from week 5 to week 8 was 65% (Table 1). The sociodemographic characteristics of the participants are shown in Table 2. Of the participants, 76.8% were female. Their mean age was 43 years old (minimum 19, maximum 77), and 76.1% had completed university-level studies. The results of the study have been made available to all participants on the website www.imcoba.es (accessed on 1 June 2021).

**Figure 1:**
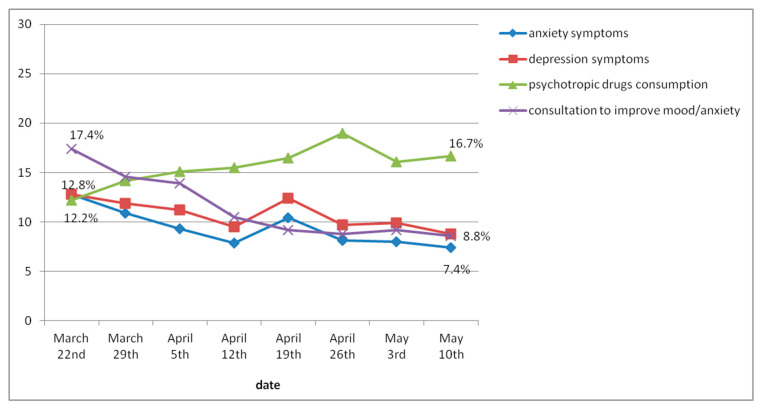
Global evolution of psychological well-being indicators.

Figure 1 shows the weekly evolution of the psychological wellbeing indicators. As the weeks of lockdown went by, the proportion of individuals with symptoms of anxiety and depression gradually decreased, except for an increase of 10.6% in anxiety symptoms and an increase of 12.4% in depressive symptoms during week 5. The proportion of participants who contacted a mental health professional or a webpage in order to improve their mood or reduce anxiety also decreased (from 17.4% to 8.6%). However, the consumption of psychotropic drugs increased from 12.2% in the first week to 16.7% in the last week.

In the first week of lockdown, 12.8% of participants presented (moderate to severe) symptoms of anxiety and 12.5% presented (moderate, moderately severe, or severe) symptoms of depression.

The changes observed in the psychological wellbeing indicators from weeks 1 to 4 and from weeks 1 to 8 are shown in Table 3. From the paired analysis, in the data, we can see a significant decrease of both anxiety and depressive symptoms after four weeks and after the entire duration of lockdown. The growth in the consumption of psychotropic drugs was significant over the first four weeks but was not significant over the entire eight-week period. Consultations aimed at improving participants’ mood or reducing anxiety decreased significantly over both the first four weeks and the whole study period.

The GEE analysis showed that the odds (OR (IC95%)) of psychological wellbeing indicators decreased every week: anxiety symptoms, 0.97 (0.96–0.98); depression symptoms, 0.98 (0.97–0.99); and consultations to improve mood or anxiety, 0.88 (0.84–0.92); except for the consumption of psychotropic drugs, which increased weekly, 1.05 (1.01–1.08). 

**Figure 2:**
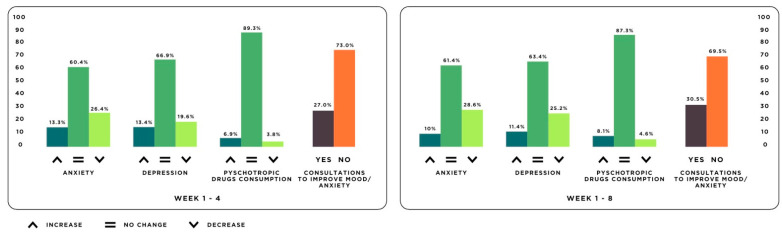
Proportion of individuals who experienced changes in anxiety, depression, and the consumption of psychotropic drugs and proportion of people who consulted to improve their mood or anxiety.

Figure 2 offers a graphical representation of the proportions of individuals who experienced changes in anxiety, depression, and the consumption of psychotropic drugs. The proportion of people who consulted to improve their mood or anxiety can also be observed. Between 60 and 70% of respondents saw no change in their levels of anxiety or depression, and 90% had no changes with regard to their consumption of psychotropic drugs. However, we do see that some participants experienced a worsened state of psychological wellbeing. After the first four weeks, 13.3% of subjects experienced increased anxiety symptoms; these figures dropped to 10% over the eight-week lockdown. Similarly, 13.4% of participants saw increased depressive symptoms from weeks 1–4, which dropped to 11.4% over the eight weeks of lockdown. As for the consumption of psychotropic drugs, 6.9% of participants increased their consumption over the first four weeks, and 8.1% increased their consumption over the entire study period. Up to 27% consulted a mental health professional or website during the first four weeks, and 30.5% did so over the whole period.

Table 4a,b shows the relationship between the sociodemographic variables and worsening psychological wellbeing indicators over the eight-week lockdown. During the first four weeks, there was a significant difference in the increase of anxiety symptoms among women (14.9% of women vs. 7.3% of men), persons aged 45 to 54 (15.0%) compared to all other ages, and those who were unemployed (30.4%). Over the entire lockdown period, the increase was still significant for women (10.4% vs. 8.5% for men), but it was those over 55 years of age and retired individuals that suffered from more anxiety (12.2%). As for depressive symptoms, during the first four weeks of lockdown, there were significantly higher levels detected among women (14.9% compared to 8.3% in men). This finding held over the entire study period (13.2% for women vs. 5.3% for men). The other variables were not found to have a significant relationship with depression symptoms. During the first four weeks, the consumption of psychotropic drugs increased significantly among participants who lived in homes with less than 80 m^2^ of floorspace (14.6%) and those who lived in homes without any outdoor space (15.1% vs. 5.3%). This significant increase held over the whole lockdown period for those who did not have space outside (16.1% vs. 6.6%). Over the first four weeks of the lockdown, women consulted healthcare professionals or websites to improve their mood or reduce anxiety significantly more than men (31% vs. 13.9%), and so did persons who found it financially difficult to reach the end of the month (31.8% vs. 23.2%). Over the entire lockdown, women continued to make more use of such services (35.2% vs. 15.2%), as did those with financial difficulties (36.5% vs. 25.8%). Neither the living situation (living alone, with children, a partner, family members, or friends) nor the level of education had an effect on psychological wellbeing.

Table 5 shows the relationship between the situational variables and changes in the indicators of psychological wellbeing indicators of participants who saw their psychological state worsen. During the first four weeks of the study, those who were worried about their jobs after the pandemic saw significant increases in anxiety symptoms (27.3% vs. 10.7%), as did those with a fear of contracting the disease (16.7% vs. 12.5%). This increase remained significant over the eight-week period for those with fear of contracting the disease (25.5% vs. 7.9%). As for depressive symptoms, though there was no significant increase over the first four weeks, those who worked from home saw a significant increase over the eight weeks of lockdown (14.2% vs. 8.3%). People worried about their work situation or with a fear of contracting the disease significantly increased symptoms of depression, both in the first four weeks (21.8% vs. 11.9% and 26.4% vs. 10.5%, respectively) and during the whole period (14% vs. 115 and 21.3% vs. 10.1%, respectively). Over the first four weeks, none of the variables considered had any significant relationship with an increased consumption of psychotropic drugs. Over the eight-week period, however, those with a fear of disease infection significantly increased their consumption of these medications (13.3% vs. 7.4%). Similarly, those worried about becoming sick made significantly more consultations to improve their mood or reduce anxiety over both the first four weeks (41.2% vs. 24.7%) and the entirety (54% vs. 28.7%) of lockdown. The other variables considered, such as interest in the evolution of the disease or the number of times that individuals went outside their homes could not be related to changes in the psychological wellbeing indicators considered.

**Figure 3:**
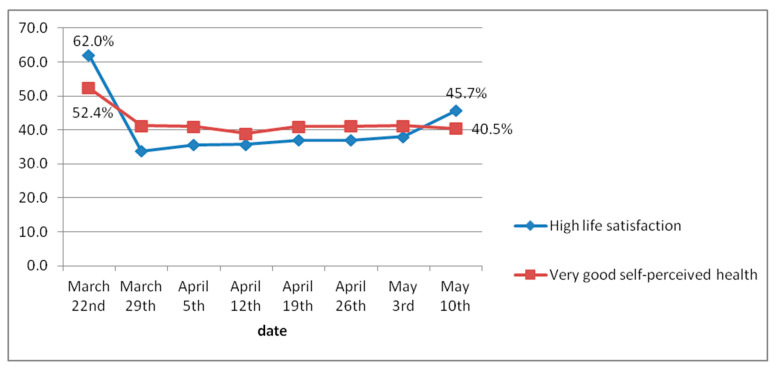
Global evolution of life satisfaction and self-perceived health.

Finally, the evolution of participants’ life satisfaction and their perception of their health in general can be seen in Figure 3. During the first week, 62% of participants said that they were very satisfied with their lives. This percentage fell to 33.7% during the second week and increased very slightly over the following weeks until reaching 41.7% in the eighth week. Individuals’ perceptions about their health followed a similar line, with 52.4% stating that they had very good health during the first week. This percentage dropped to 41.2% during the second week and ended up at 40.5% during the last week of lockdown.

Participants seem to have been more optimistic about their personal futures (with a mean value of 3.1 out of 5) than the future of society in general (with a mean of 2.4 out of 5). A total of 33.4% of the respondents claimed to be optimistic or very optimistic about their personal futures, while only 10.9% felt the same levels of optimism about the future of society. Women were significantly less optimistic than men, both in terms of personal futures (20.7% vs. 42.3%) and the future of society in general (9.0% vs. 17.5%). Individuals under 35 years of age were the least optimistic about their futures. There were no statistical differences by age regarding optimism about the future of society.

## 4. Discussion

The present study provides the results of an assessment of the psychological wellbeing of a population of adults consisting of 681 residents in the Balearic Islands over Spain’s eight-week lockdown. Despite the initially high levels of depressive and anxiety symptoms, as well as consultations made to improve individuals’ mood and reduce anxiety, these levels decreased significantly over lockdown (which took place from 15 March to 10 May). Nevertheless, the consumption of psychotropic drugs increased over the period. We can see that some segments of the population showed worse indicators of psychological wellbeing, namely, women, those aged over 45, and persons worried about their jobs after the pandemic. Persons worried about contracting COVID-19 were those who saw their mental health suffer the most, as they saw increased depressive and anxiety symptoms, consumed more psychotropic drugs, and had more mental health consultations over the study period. Other indirect indicators of psychological wellbeing, such as life satisfaction, self-perceived state of health, and optimism regarding one’s personal future and the future of society all declined over lockdown.

Quarantines that have been put in place during previous pandemics, such those caused by SARS, Ebola, or the H1N1 virus, had negative repercussions on the psychological wellbeing of those affected, and these repercussions lasted for months after lockdowns were over [4]. In our study, the results show a negative impact on the psychological wellbeing of the population during the first week of lockdown, as the mean values of anxiety and depression symptoms reported in the National Health Survey (Encuesta Nacional de Salud) were almost doubled (12.8% vs. 6.7%) [18]. However, the values we found regarding the initial proportions of depressive and anxiety symptoms were well below, and in some cases half of, those seen in some cross-sectional studies carried out in Spain and other countries’ [19,20,21,22,23,24,25,26,27,28] anxiety symptoms have been reported to affect between 25% and 45% of the population and depressive symptoms between 14% and 45% of the population. The proportion of persons who claimed to consume psychotropic drugs in our study was greater than the proportion provided by the National Health Survey (12% vs. 11%) [18]. Additionally, we found that the proportion of respondents who reported consulting a healthcare professional in order to improve their mood or reduce anxiety was greater in our study than the proportion of respondents to the National Health Survey who reported visiting a psychologist or psychotherapist (17% vs. 5%). These results are in line with the results of Beck et al. [29], who observed an increase in the consumption of psychotropic medications for sleeping: 16% of individuals reported taking psychotropic medications in the last 12 months, and of these, 41% did so after lockdown.

With regard to the psychological wellbeing evolution over the eight-week study period, and in contrast to our initial hypothesis, we saw a progressive improvement in individuals’ depressive and anxiety symptoms. The upticks that occurred in anxiety and depressive symptoms in week 4–5 could be related to different contextual aspects, because in Spain, all non-essential activities were paralyzed; the state of alarm was prolonged by the government; on 2 April, the record of deaths from the coronavirus was broken (950 people); the daily contagion cases increased exponentially (an average of 7000 people a day and rising), and on 9 April, the barrier of 150,000 people counted was exceeded. All these factors could probably explain the worsening in psychological scores, by overcoming the ability of people to assimilate all this negative information at once. After week 4–5, there was probably an effect of acceptance and/or habituation to the new situation. The increase in the consumption of psychotropic drugs could also be related to the improvement of psychological symptoms. Other studies have reported a similar evolution of symptoms, with descriptions of a strong initial impact that decreases significantly or shows no significant changes after four to six weeks of living with lockdown measures [28,30,31,32,33,34]. Along this line, a systematic review carried out by Prati et al. [35] of 25 longitudinal studies on the effects of confinement found a low level of psychological impact on the population, with small but significant effects on levels of anxiety and depression. These data suggest that the effects of lockdowns are not purely negative, and that many people are psychologically resilient to their effects [25,36,37,38,39]. This could be related to differences in the socio-cultural contexts faced by different populations and/or differences between countries. However, other longitudinal studies carried out in Spain have reported a significant increase in depressive symptoms, both between subjects and within subjects, during the first weeks of lockdown, though there is no consensus regarding the progression of anxiety [34,40,41]. The differences between these studies could be due to the different scales they used to measure anxiety and depression, their cut-off points, their design, or the range of periods considered. Cross-sectional studies, on the other hand, have widely ranging data-collection and inclusion periods (varying on the order of weeks), which makes interpreting their results more difficult, as the impact on participants’ psychological state changes from week to week. Another reason for these observed differences may lie in the situation experienced in the Balearic Islands during the first wave of the pandemic, with COVID-19 infection and mortality rates lower than those seen in the rest of Spain and other countries [42]. This may have led to lower levels of anxiety, depression, and worry about the disease. Along this line, Fitzpatrick et al. [43] found that the fear of contracting COVID-19 was greater in regions of the United States of America that were more affected by the pandemic and that this fear ran parallel to psychological distress.

Unlike anxiety and depression symptoms, the consumption of psychotropic drugs increased throughout the lockdown, starting at 12.2% of the population and finishing at 16.7%. Those who saw their levels of anxiety and depression reduced were not seen to have a greater level of consumption of psychotropic drugs than other participants. The use of psychoactive or psychotropic substances during lockdown is a subject that has been studied by the French Society of Pharmacology and Therapy (Société Française de Pharmacologie et de Thérapeutique) [44] and other agencies that monitor drug use and addiction [45], and they describe significant increases. Furthermore, the study by Beck et al. [29] found an increase in the consumption of psychotropic drugs for sleeping during lockdown. According to these authors, doctors and psychiatrists may have been more likely to prescribe these medications to their patients than recommend them non-pharmacological treatments.

Despite the favorable evolution of anxiety and depression levels, we found that the impact of lockdown on other indicators, such as life satisfaction and self-perceived state of health, was greater. During the first week of lockdown, high levels of life satisfaction and perceived good health were cut nearly in half, and they gradually decreased over the following weeks. Many authors have focused their efforts on studying the impact of COVID-19 on levels of life satisfaction during lockdown, in addition to other possibly related factors [46,47]. In our study, though we address this aspect with a single overall question, the results obtained coincide with those from the aforementioned studies that use multidimensional scales. In their review, Prati et al. [35] found no evidence that lockdown affected wellbeing or life satisfaction, but their results should be viewed with some caution, as the size of the effect was small *(g* = 0.17), had large confidence intervals, and there was a high level of heterogeneity, which the authors attribute to some of the methodological factors of the studies they considered [38].

In our study, women suffered higher levels of psychological distress (i.e., symptoms of anxiety and depression) and made more consultations to improve their mood, a finding that is mirrored in various other studies [6,7,19,20,28,46,48]. Additionally, they were less optimistic about the future, both on a personal level and with regard to society in general. These results may reflect gender differences that still persist in our society, where women continue to take on the role of caregivers, and thus, they must combine work and household chores, while their work–life balance suffers [49,50,51,52].

Those over the age of 45 saw increased levels of symptoms of anxiety, a finding that is in line with the results obtained by Bavel [53], who highlights that the psychological wellbeing of older individuals is more susceptible to worsening in social isolation. However, our study shows that it was individuals under 35 years old who were less optimistic about their personal futures. These results are consistent with those seen in various other studies that have found that younger persons were more affected psychologically during the pandemic, perhaps, as some authors point out, because their daily routines suffered more, they were hit harder economically, or because they had fewer resources on a personal or cognitive level [27,33,48,54,55].

Individuals without a job (either unemployed or not actively working) and those working from home had more symptoms of anxiety compared to those who worked on-site. Additionally, those worried about their jobs experienced more symptoms of anxiety and depression, a finding in accordance with other studies that have described individuals’ employment status as a factor that determines changes in psychological wellbeing [19,21,54,56].

People who found it more financially difficult to reach the end of the month made significantly more consultations to mental health professionals and websites to improve their mood/reduce anxiety. Having a worse socioeconomic status is associated with worse psychological wellbeing during lockdown, as has been shown in recent studies [7,23,57]. Despite the excellent housing conditions of the majority of the participants in our study, those with dwellings that did not have any outdoor space (e.g., a balcony, terrace, or garden) and those who lived in homes with less than 80 m^2^ saw significantly increased levels of psychotropic drug consumption. Other authors have also described that worse housing conditions may result in psychological distress and mental health issues [20,58].

One of the elements considered that has influenced all the dimensions of psychological wellbeing is fear of disease infection. Those who were more worried about contracting COVID-19 were those who suffered the most negative impact on their psychological wellbeing, as they saw significantly increased symptoms of anxiety and depression, consumption of psychotropic drugs, and consultations to improve their mood/reduce anxiety. Jaques-Avinyó et al. [20] also found a positive relationship between anxiety symptoms and the fear of being infected by COVID-19. Some other studies [4,53] that measured this variable did not assess its relationship with psychological wellbeing.

Our study has meaningful strengths. It is one of the few longitudinal studies that has assessed the weekly progression of psychological wellbeing over the entirety of lockdown, a fact that allows us to shed light on how mental health changes during such a unique situation. Many other studies, in contrast, take a cross-sectional approach to considering mental health, or they take only two measurements. Another strength is that, despite making use of an online survey, we achieved a high response rate over the entire study period. We made use of valid measurements as well as other, more subjective ones, such as optimism and life satisfaction, which allowed us to consider participants’ general mood in the face of this unusual situation. One part of our analysis focused on individuals who suffered from negative consequences of lockdown and the epidemic. Although these people were a minority in our sample, they allowed us to identify which aspects can be improved upon in the future to alleviate the possible negative effects stemming from a lockdown.

Our study also has some limitations that are worth mentioning. First, we did not know the psychological wellbeing of participants before lockdown, but we can compare baseline data with the data available from the 2017 National Health Survey. This does not allow us to measure the actual size of the impact, an issue that comes up in some of the other studies mentioned herein [9]. However, as we conducted a longitudinal study with weekly assessments, we were able to study the progression of psychological wellbeing throughout the pandemic. The increase in anxiety and depression symptoms between the weeks studied was measured on the basis of the change in severity category established in each scale (i.e., from mild to moderate), without taking into account whether this change was clinically relevant. However, we do not consider this to be a relevant limitation since the study was conducted in a mostly asymptomatic population rather than a clinical setting. Secondly, the selection method used (snowball sampling) and the online surveys might call into question the representativeness of the sample. As with other studies [19,20,59], those who responded to our survey were mostly women, highly educated, with jobs, and had very favorable living conditions. This means that some groups were excluded, groups that, because of their socioeconomic situation, may have suffered greater mental distress during lockdown. Having a more diverse sample, as mentioned in the study by O’Connor et al. [31], would help in achieving greater representativeness and would provide more realistic measurements. Thirdly, and along this same line, individuals who do not use digital technologies were under-represented, an issue that might also undermine the external validity of our findings.

## 5. Conclusions

Lockdown had an initially negative impact on the psychological wellbeing of the participants in this study. However, the evolution of depressive and anxiety symptoms, as well as the number of consultations made to better participants’ mood/reduce anxiety, improved as the weeks went by. This was not the case for the evolution of the consumption of psychotropic drugs, life satisfaction, or self-perceived health, all of which worsened over the study period. Optimism over personal futures and the future of society in general, measured during the final week, was also impacted negatively. Our findings provide evidence that the individuals most vulnerable to the effects of lockdown and those who reported the most negative effects in terms of some of the indicators of psychological wellbeing were women, those over 45 years old, those who worked from home, those who were unemployed or retired, those worried about their jobs, and those living in a dwelling with less than 80 m^2^ or with no outdoor space. Being fearful of contracting COVID-19 was the only factor that was associated with all of the dimensions of psychological wellbeing. Our findings highlight the importance of supporting people in the period before future lockdowns, thus reducing distress, perhaps by providing more information to reduce excessive fears about becoming sick. More studies that include other strata of the population are needed in order to better understand the impact that lockdowns have on those who are most vulnerable and who have worse living conditions, as the sample in our study is not representative of the general population.

## Figures and Tables

**Table 1:** Participation during lockdown.

Date	22 March	29 March	5 April	12 April	19 April	26 April	3 May	10 May
*n* participants	681	485	489	522	459	456	439	421
Percentage	100%	71.2%	71.8%	76.6%	67.4%	66.9%	64.4%	61.8%

**Table 2:** Participant characteristics.

	*n*	%
	681	
Sex		
Women	523	76.8
Men	158	23.2
Age		
<35 years	184	27.3
35–44 years	200	29.6
45–54 years	142	20.9
≥55 years	149	22.1
Education		
Primary education	22	3.2
Secondary education	140	20.7
Higher education	515	76.1
Employment status		
Employed	514	75.5
Unemployed	35	5.1
Retired	50	7.3
Others	80	11.7
Concern about employment status after lockdown (week 1)		
Little concern	388	75.6
High concern	125	24.4
Working conditions during lockdown (week 1)		
On-site work	103	20.2
Work from home	296	58.0
On-site work + work from home	111	21.8
Monthly income, difficulty to reach the month’s end		
With difficulty	318	48.3
Without difficulty	341	51.7
Live with		
Alone	85	12.5
With a partner	165	24.2
With a partner and children	269	39.5
With partner and children and parents	16	2.3
With children without another adult	32	4.7
With parents	66	9.7
Sharing with other family/friends	38	5.6
Others	10	1.5
Household characteristics		
Not outdoor space	97	14.3
With outdoor space	583	85.7
Household size		
<80 m^2^	137	20.1
80–100 m^2^	191	28.1
>100 m^2^	352	51.8
Life satisfaction (1st week)		
Low	22	3.2
Moderate	236	34.8
High	421	62
Self-perceived health (1st week)		
Bad/regular	65	9.5
Good	259	38
Very good/excellent	357	52.4

**Table 3:** Evolution of psychological wellbeing indicators.

	*n* = 681	Week 4 *n* = 522	Week 1–4 Difference %	*p* *	Week 8 *n* = 421	Week 1–8 Difference %	*p* *
	*n* (%)	*n* (%)			*n* (%)		
Gad-7 scale							
No symptoms	379 (59.5)	357 (69.5)	10	<0.001	314 (74.9)	15.4	0.000
Mild symptoms	176 (27.6)	116 (22.6)	−5		74 (17.7)	−9.9	
Moderate symptoms	57 (8.9)	28 (5.4)	−3.5		23 (5.5)	−3.4	
Severe symptoms	25 (3.9)	13 (2.5)	−1.4		8 (1.9)	−2.0	
PHQ-9 scale							
No depression/minimal	394 (61.6)	348 (67.6)	6	0.027	306 (73.0)	11.4	0.000
Mild depression	164 (25.6)	118 (22.9)	−2.7		76 (18.1)	−7.5	
Moderate depression	59 (9.2)	32 (6.2)	−3.0		25 (6.0)	−3.2	
Moderately severe	14 (2.2)	13 (2.5)	0.3		11 (2.6)	0.4	
Hard depression	9 (1.4)	4 (0.8)	−0.6		1 (0.2)	−1.2	
Psychotropic drugs consumption				0.040			0.070
No	570 (87.8)	436 (84.5)	−3.3		348 (83.3)	−4.5	
Yes	79 (12.2)	80 (15.5)	3.3		70 (16.7)	4.5	
Consultations to improve mood or anxiety				0.006			
No	537 (82.6)	462 (89.5)	6.9		383 (91.4)	8.8	0.000
Yes, to a professional	37 (5.7)	21 (4.1)	−1.6		16 (3.8)	−1.9	
Yes, on a website	76 (11.7)	33 (6.4)	−5.3		20 (4.8)	−6.9	

*p* * McNemar-Bowker test.

**Table 4:** Relationship between the sociodemographic variables and worsening psychological wellbeing indicators over the eight-week lockdown.

(a)								
	Increased Anxiety Symptoms (GAD-7)				Increased Depression Symptoms (PHQ-9)			
	Week 1–4	*p* *	Week 1–8	*p* *	Week 1–4	*p* *	Week 1–8	*p* *
**Sex**		0.030		0.004		0.021		0.020
Women	58 (14.9)		32 (10.4)		58 (14,9)		41 (13.2)	
Men	8 (7.3)		8 (8.5)		9 (8,3)		5 (5.3)	
**Age**		0.010		0.020		0.310		0.212
<35 years	18 (14.3)		11 (10.9)		20 (15.9)		12 (11.8)	
35–44 years	16 (10.5)		7 (6.5)		17 (11)		13 (12)	
45–54 years	17 (15)		11 (11.1)		16 (14.2)		12 (12.1)	
>55 years	15 (14.7)		11 (12.2)		14 (13.7)		9 (9.9)	
**Education**		0.840		0.924		0.948		0.425
No university studies	16 (14.2)		9 (9.1)		16 (14.2)		10 (10)	
With university studies	50 (13.2)		31 (10.3)		50 (13.1)		36 (12)	
**Employment situation**		0.003		0.017		0.065		0.178
Employed	41 (10,8)		30 (9.6)		47 (12,3)		35 (11.2)	
Unemployed	7 (30.4)		3 (17.6)		3 (13)		2 (11.8)	
Retired	8 (23.5)		6 (21.4)		7 (20.6)		6 (20.7)	
Others	10 (17.5)		1 (2.2)		10 (17.5)		3 (6.7)	
**Economic difficulty at the end of the month**		0.595		0.106		0.075		0.455
With difficulty	34 (14.7)		24 (13)		39 (16.8)		24 (13)	
Without difficulty	31 (12.1)		14 (6.8)		26 (10.1)		20 (9.6)	
**Housing characteristics**		0.255		0.633		0.833		0.660
Not outdoor space	9 (12,3)		8 (12.9)		11 (15.1)		9 (14.5)	
With outdoor space	56 (13.2)		31 (9.1)		55 (12.9)		36 (10.6)	
**Housing size**		0.306		0.993		0.112		0.831
<80 m^2^	19 (20)		7 (9.1)		20 (21.1)		11 (14.3)	
80–100 m^2^	16 (11.4)		11 (9.8)		17 (12.1)		12 (10.5)	
>100 m^2^	31 (11.9)		22 (10.3)		30 (11.5)		23 (10.8)	
**(b)**								
	**Increased Consumption of Psychotropic Drugs**				**Consultations to Improve Mood/Anxiety**			
	**Week 1–4**	*p* *	**Week 1–8**	*p* *	**Week 1–4**	*p* *	**Week 1–8**	*p* *
**Sex**		0.212		0.063		<0.001		<0.001
Women	30 (7.7)		28 (9.0)		162 (31,0)		184 (35.2)	
Men	5 (4.5)		5 (5.2)		22 (13.9)		24 (15.2)	
**Age**		0.782		0.735		0.140		0.216
<35 years	7 (5.6)		6 (5.8)		55 (29.9)		61 (33.2)	
35–44 years	9 (5.9)		9 (8.4)		54 (27.0)		56 (28.0)	
45–54 years	12 (10.4)		9 (8.9)		44 (31,0)		51 (35.9)	
>55 years	7 (6.5)		9 (9.5)		30 (20,1)		39 (26.2)	
**Education**		0.517		0.350		0.806		0.596
No university studies	11 (9.3)		9 (8.7)		45 (27,8)		52 (32.1)	
With university studies	24 (6.3)		24 (7.9)		138 (26.8)		154 (29.9)	
**Employment situation**		0.894		0.422		0.177		0.152
Employed	26 (6.8)		27 (8.7)		144 (28.0)		164 (31.9)	
Unemployed	1 (4.2)		1 (5.6)		7 (20.0)		7 (20.0)	
Retired	3 (7.9)		4 (12.1)		8 (16.0)		10 (20.0)	
Others	5 (8.9)		1 (2.2)		25(31.2)		27 (33.8)	
**Economic difficulty at the end of the month**		0.193		0.896		0.013		0.003
With difficulty	21 (9.1)		14 (7.7)		101 (31.8)		116 (36.5)	
Without difficulty	13 (5)		18 (8.4)		79 (23.2)		88 (25.8)	
**Housing characteristics**		0.009		0.028		0.226		0.192
Not outdoor space	11 (15.1)		10 (16.1)		31 (22.0)		35 (36.1)	
With outdoor space	23 (5.3)		23 (6.6)		152 (26.1)		172 (29.5)	
**Housing size**		0.018		0.778		0.297		0.243
<80 m^2^	14 (14.6)		8 (10.3)		44 (32.1)		50 (36.5)	
80–100 m^2^	9 (6.4)		9 (7.9)		47 (24.6)		56 (29.3)	
>100 m^2^	12 (4.5)		16 (7.4)		93 (26.4)		102 (29.0)	

*p* * chi-squared test.

**Table 5:** Relationship between the situational variables and worsening psychological wellbeing indicators over the eight-week lockdown.

(**a**)								
	**Increased Anxiety Symptoms (GAD-7)**				**Increased Depression Symptoms (PHQ-9)**			
	**Week 1–4**	*p* *	**Week 1–8**	*p* *	**Week 1–4**	*p* *	**Week 1–8**	*p* *
**Working conditions during lockdown**		0.657		0.533		0.235		0.014
Work from home	20 (10.1)		17 (11.0)		22 (11.0)		22 (14.2)	
Others	21 (11.5)		13 (8.3)		25 (13.7)		13 (8.3)	
**Concern about employment status after**		<0.001		0.111		0.001		0.021
lockdown								
Little concern	45 (10.7)		30 (8.7)		50 (11.9)		38 (11.0)	
High concern	21 (27.3)		10 (17.5)		17 (21.8)		8 (14.0)	
**Fear of COVID-19 infection**		0.020		<0.001		0.000		0.050
Little fear	51 (12.5)		28 (7.9)		43 (10.5)		36 (10.1)	
High fear	15 (16.7)		12 (25.5)		24 (26.4)		10 (21.3)	
**Times out during lockdown**		0.746		0.064		0.957		0.408
Rarely out	43 (14.1)		22 (12.9)		42 (13.7)		21 (12.3)	
Frequently out	23 (12.0)		18 (7.8)		25 (13)		25 (10.7)	
**Interest in COVID-19 evolution**		0.133		0.191		0.229		0.893
Little interested	33 (13.1)		23 (8.5)		28(11.1)		30 (11.0)	
Very interested	33 (13.5)		17 (13.0)		39 (15.9)		16 (12.2)	
(**b**)								
	**Increased Consumption of Psychotropic Drugs**				**Consultations to Improve mood/Anxiety**			
	**Week 1–4**	*p* *	**Week 1–8**	*p* *	**Week 1–4**	*p* *	**Week 1–8**	*p* *
**Working conditions during lockdown**		0.306		0.952		0.918		0.388
Work from home	13 (6.5)		13 (8.4)		60 (27.3)		51 (28.0)	
Others	13 (7.1)		14 (8.9)		127 (26.9)		157 (31.5)	
**Concern about employment status after**		0.188		0.602		0.141		0.281
lockdown								
Little concern	27 (6.4)		28 (7.9)		156 (26.1)		186 (30.0)	
High concern	8 (10.1)		5 (8.9)		28 (33.7)		22 (36.7)	
**Fear of COVID-19 infection**		0.256		0.029		0.001		<0.001
Little fear	25 (6.1)		27 (7.4)		144 (24.7)		181 (28.7)	
High fear	10 (10.9)		6 (13.3)		40 (41.2)		27 (54.0)	
**Times out during lockdown**		0.853		0.362		0.531		0.644
Rarely out	20 (6.5)		13 (7.6)		89 (28.2)		63 (31.8)	
Frequently out	15 (7.7)		20 (8.4)		95 (26.0)		145 (30.0)	
**Interest in COVID-19 evolution**		0.545		0.284		0.980		0.944
Little interested	15 (5.9)		18 (6.6)		112 (27.1)		160 (30.5)	
Very interested	20 (8.0)		15 (11.1)		72 (27.0)		48 (30.8)	

*p* * chi-squared test.

## Data Availability

The data presented in this study are available on request from the corresponding author.
